# Regulation of cell polarity determinants by the Retinoblastoma tumor suppressor protein

**DOI:** 10.1038/srep22879

**Published:** 2016-03-14

**Authors:** Sandhya Payankaulam, Kelvin Yeung, Helen McNeill, R. William Henry, David N. Arnosti

**Affiliations:** 1 Department of Biochemistry and Molecular Biology, Michigan State University, East Lansing, MI 48824, USA; 2 Lunenfeld- Tanenbaum Research Institute, Mount Sinai Hospital, 600 University Avenue, Room 881, Toronto, Ontario M5G1X5, Canada

## Abstract

In addition to their canonical roles in the cell cycle, RB family proteins regulate numerous developmental pathways, although the mechanisms remain obscure. We found that Drosophila Rbf1 associates with genes encoding components of the highly conserved apical–basal and planar cell polarity pathways, suggesting a possible regulatory role. Here, we show that depletion of Rbf1 in *Drosophila* tissues is indeed associated with polarity defects in the wing and eye. Key polarity genes *aPKC*, *par6*, *vang*, *pk*, and *fmi* are upregulated, and an *aPKC* mutation suppresses the Rbf1-induced phenotypes. RB control of cell polarity may be an evolutionarily conserved function, with important implications in cancer metastasis.

The Retinoblastoma tumor suppressor protein (RB) is a conserved central regulator of eukaryotic cell cycle, a function that has been extensively documented^1^ (and references therein). RB restricts entry of cells into S phase through its activity as a corepressor of the E2F family of transcription factors. RB activity is functionally downregulated during cell cycle progression by G1 Cyclin/CDKs, whose activities result in extensive phosphorylation and allosteric changes that inactivate RB^2^. Steady-state levels of RB family members, including the p107 and p130 homologs, are additionally regulated at the level of protein stability via the ubiquitin-proteosome pathway^3^. In addition to well-characterized roles in cell cycle progression, RB family proteins play key roles in differentiation, apoptosis, genome stability, and senescence, although it is less well understood how RB family proteins influence these additional biological pathways^4^,^5^,^6^.

Essential features of RB regulation are recapitulated in the Drosophila system, which features two members, Rbf1 and Rbf2, providing a streamlined model to interrogate Rbf-E2F roles in developmental programs^7^. In genome-wide studies, we found that Rbf1 occupies a number of promoters for key genes linked to conserved cellular signaling pathways, including the insulin, Hippo, Jak /Stat and polarity pathways, in addition to canonical cell cycle genes^8^. Thus, the impact of RB proteins on diverse developmental processes may in part reflect evolutionarily conserved transcriptional regulation of signaling components. Indeed, Rbf1 and the Hippo tumor suppressor pathway are functionally linked during photoreceptor differentiation. In genetic tests, strong synergy was noted between *rbf1* and the Hippo pathway kinase *warts*, resulting in increased proliferation, suppression of apoptosis and a failure of photoreceptor differentiation^9^. An RB-Hippo link has also been noted in mammals; partial knockdown of LATS2 compromises phosphorylated RB’s ability to suppress cell proliferation and induction of senescence in Saos2 cells, and triple knockout liver cells lacking RB and its homologs p107 and p130 showed elevated expression of the Hippo pathway transcriptional effector YAP, as well as YAP target genes^10^,^11^. Molecular studies have also linked RB to the insulin and mTOR signaling pathways^12^,^13^. Collectively, these studies suggest that RB plays a pivotal and evolutionarily conserved role in regulation of core signaling genes with pleiotropic roles in cell growth.

A large proportion of promoters for polarity and planar cell polarity (PCP) genes are bound by Rbf1, much more than would be expected by chance (Chi square test P < 0.001), raising the question of whether the corepressor might regulate polarity pathways^14^,^15^. Apical/basal polarity, a characteristic of all epithelial cells, allows for specialized functions such as directional transport of ions and regulation of asymmetric cell divisions. Proper establishment of apical/basal polarity is key to maintaining normal tissue architecture and tissue homeostasis, and provides a tumor suppressive function as a block against malignant invasion^16^. In addition, some epithelial cells display planar cell polarity, in which the vector for polarization is orthogonal to the epithelial plane. At a macroscopic level, the regimented orientation of scales in fish, feathers in birds and hairs in mammals are examples of PCP^17^. Much of our understanding of the genetics of this evolutionarily conserved process originates from studies in Drosophila, where PCP is visualized as distally pointing hairs on the wing, posterior pointing hairs on the abdomen and in the stereotypic orientation of ommatidia in the eye. Polarity genes show dynamic stage- and tissue-specific regulation, however little is known about mechanisms controlling transcription levels of these genes. Here, we report evidence for direct functional links between Rbf1 and conserved polarity genes, providing a novel molecular connection between regulation of cell cycle and cell polarity.

## Results

### Rbf1 depletion induces strong polarity defects in eyes and notum

**Figure 1:**
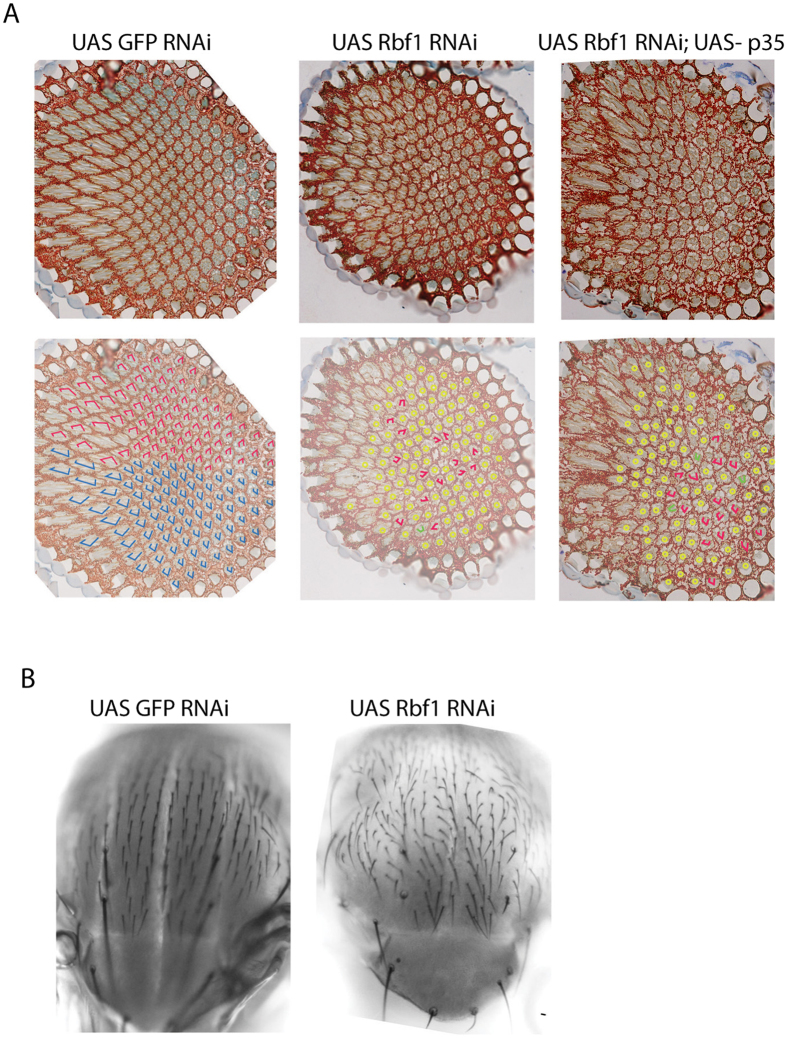
Depletion of Rbf1 induces polarity defects in eye and notum. (**A**) Tangential ommatidial sections were analyzed for orientation and number of photoreceptor cells in individual ommatidia. Control eyes showed normal number and dorsal/ventral orientation of cell clusters (duplicate eye section marked below original image; red trapezoids show dorsal and blue show ventral orientations). Rbf1 RNAi (center) and Rbf1 RNAi/UAS p35 (right) displayed similar phenotypes; ommatidia exhibited randomly oriented groups of photoreceptor cells (red), as well as many ommatidia lacking the full complement of cells (yellow). A few ommatidia with full complement of photoreceptor cells showed a symmetrical rather than trapezoidal arrangement (green). Anterior is to the right. 400 to 1000 ommatidia were analyzed for polarity defects in multiple sections from four to ten individuals. No equatorial midline could be defined either in Rbf1 RNAi or in Rbf1 RNAi/UAS p35 sections. (**B**) Bristle orientation defects seen on notum of Rbf1 RNAi individual (right panel). Anterior is to the top. *ey-Gal4* was used for expression in the eye, and *pen*-*Gal4* for expression in the notum. Similar but generally weaker phenotypes were observed with the *GMR-Gal4* driver. The results shown were generated using Rbf1 RNAi line obtained from the Bloomington Stock Center; similar but overall weaker phenotypes were observed with a line obtained from VDRC (data not shown).

The establishment of polarity in different tissue types commonly involves several core genes, including *fz, dsh, pk, vang, dgo and fmi.* To understand the biological relevance of Rbf1 interaction with cell polarity gene promoters, we tested whether depletion of Rbf1 phenocopies polarity defects, such as those observed in *fz*, *vang*, and other core polarity mutants^18^. In flies, polarity can be visualized in tissues of epithelial origin such as the eye, wing, and thorax. In the eye, planar polarity is apparent in the orderly arrangement of the ommatidia in the dorsal/ventral axis. Misrotation and loss of photoreceptor cells within the ommatidia were observed upon loss or gain of function of apical/basal polarity determinants^19^. We used the Gal4-UAS system to deplete Rbf1 in the eye and assayed for polarity defects. Analysis of tangential sections revealed that Rbf1 RNAi generated two phenotypes that showed disruption of photoreceptor morphology, and was apparent in all the eyes analyzed (Fig. 1A). First, in ommatidia with the full complement of photoreceptor cells, a specific polarity phenotype was apparent, whereby the eight cells were canonically disposed, but their rotation and chirality was random, similar to the phenotype of PCP mutants^20^. These rotation defects may reflect Rbf1 regulation of core polarity genes, and/or regulation of another Rbf1 target gene *canoe*, which is also physically bound by Rbf1. *canoe* is an effector of the EGFR pathway, important for regulating ommatidial rotation in the eye^21^,^22^. Second, a significant number of ommatidia showed fewer than eight photoreceptors. Consistent with this observation, earlier studies also found that *rbf1* clones display disorganized ommatidia lacking one or more photoreceptors and cone cell defects^23^. The lack of photoreceptor cells may represent Rbf1 acting through polarity determinants as well as through control of genes involved cell differentiation and proliferation. Similar results were obtained with two different *UASRbf1 RNAi* lines, with stronger phenotypes observed using the Bloomington line shown in Fig. 1A. We observed similar phenotypes with *ey-Gal4* and *GMR-Gal4*; combining the *ey-Gal4* and *GMR*-*Gal4* drivers produced a more severe phenotype (data not shown). We did not note polarity defects in eyes of *rbf*^ 120/14Δ^ heterozygotes, presumably because this reduction is less severe than in the RNAi background.

During eye development, in early third instar larval stages, the actual determination and specification of photoreceptor cells begin in the morphogenetic furrow (MF); loss of Rbf1 results in a pattern of cell death visualized in the morphogenetic furrow^24^,^25^. In order to understand if Rbf1 RNAi phenotypes in the eye are secondary to apoptosis, we coexpressed the p35 apoptotic inhibitor along with Rbf1 RNAi and analyzed polarity defects. p35 expression alone did not affect ommatidial architecture; the eyes showed mostly normal architecture, with a few ommatidia showing loss of photoreceptors (less than 5%, data not shown). However, we did not observe suppression of polarity defects when Rbf1 RNAi was carried out in this background. We observed similar loss of photoreceptors (84% of ommatidia affected) and of the ommatidia with full complement of photoreceptor cells, all showed orientation defects, suggesting that Rbf1 induced polarity defects are independent of apoptosis (Fig. 1A). We also depleted Rbf1 levels in the presumptive notum using the *Pen-Gal4* driver. In these flies, defects were observed in the thoracic bristle pattern, whereby the normal anterior-posterior bristle orientation was completely disrupted on the notum, similar to the phenotype previously described for *fz* and *fmi* mutants^26^ (Fig. 1B).

### Rbf1 depletion-induced polarity defects in Drosophila wings

There are significant tissue-specific differences in the requirement for some polarity genes, as well as in the specific downstream effectors needed for establishing polarity. In the wing, polarity is established largely independently of *ft* and *ds*, whereas these genes are essential for polarity in the eye^27^. Additional differences involve downstream effectors of polarity proteins; *mwh*, *fy* and *in* serve as downstream effectors in the wing whereas *Delta* serves as a downstream effector of *fz* in the eye, inducing Notch signaling in neighboring cells^28^,^29^. We therefore analyzed the effects of Rbf1 knockdown in developing wings.

**Figure 2:**
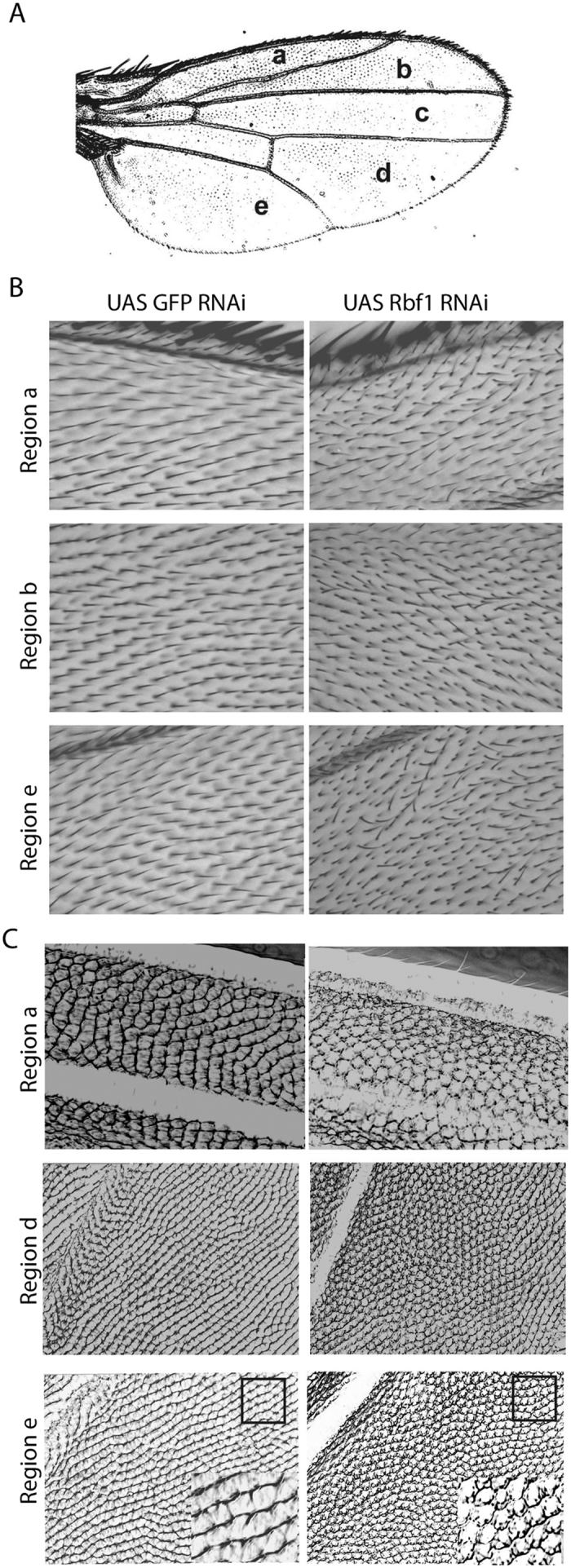
Rbf1 RNAi causes polarity defects in Drosophila wings. (**A**) Schematic representation of regions of adult wing as noted in Wong and Adler^18^. (**B**) Bristle orientation. Representative regions of wings showing orientation of wing hairs. In contrast to the normal proximal-distal orientation in control flies (left panels), Rbf1 RNAi expressing flies showed misorientation of wing hairs (right panels). The wings are oriented distal to the right, anterior to the top. (**C**) Ridge assay. To measure longitudinal ridges formed by edges of closely packed hexagonal cells, wild-type and Rbf1 RNAi wings were imaged as described^32^. Anterior regions (a) show normal A-P ridge orientation, while posterior regions (d,e) show P-D orientation in control wings (left panels). No clear ridge formation is apparent in Rbf1 RNAi expressing flies (right panels). Inserts in lower panels show magnified images to illustrate change in cell shape observed in Rbf1 RNAi wing. More than one hundred wings were analyzed for polarity and ridge defects. *pen* > Gal4 was used for expression in the wing. The results shown were generated using Rbf1 RNAi line obtained from the Bloomington Stock Center. Similar phenotypes were observed with a *Bx > Gal4* driver; effects were restricted to posterior wing compartment using *en-Gal4*.

Rbf1 RNAi resulted in subtle but reproducible wing hair polarity defects in all flies, manifested by the loss of highly stereotypic proximal–distal orientation of hairs in several sectors of the adult structure (Fig. 2A,B). Unlike the pleiotropic effects on all wing hairs seen in a *fz* null background, here we observed multiple, isolated sectors of disrupted polarity on affected wings.

We additionally analyzed the orientation of adult wing cuticular ridges, prominent aligned edges of cells that extend across the wing. The formation of ridges reflects polarized cellular organization. The development of ridges requires planar cell polarization and hexagonal cell packing, and as with hair polarity, this process is regulated by the PCP signaling pathway, although with some differences^30^,^31^,^32^. Ridges are oriented anterior-posterior in the wing anterior, and proximal-distal in the wing posterior regions. Rbf1 RNAi wings showed greatly reduced ridge structure across the entire surface of the wing, both in the anterior and posterior regions (Fig. 2C). This phenotype is similar to the defect observed in *pk* mutants^32^. In addition, cells in Rbf1 RNAi wings exhibited a “cobblestone” pattern indicating irregular packing, a process which is influenced by PCP components^31^. To mitigate RNAi off target effects, we tested the two independent RNAi lines with various wing specific drivers for polarity defects. We observed similar hair orientation and cuticular defects using an alternative *UASRbf1 RNAi* line as well as alternative Gal4 drivers (*en-Gal4* and *Beadex-Gal4*; data not shown).

### Rbf1 represses transcription of key polarity genes in wing imaginal discs

**Figure 3:**
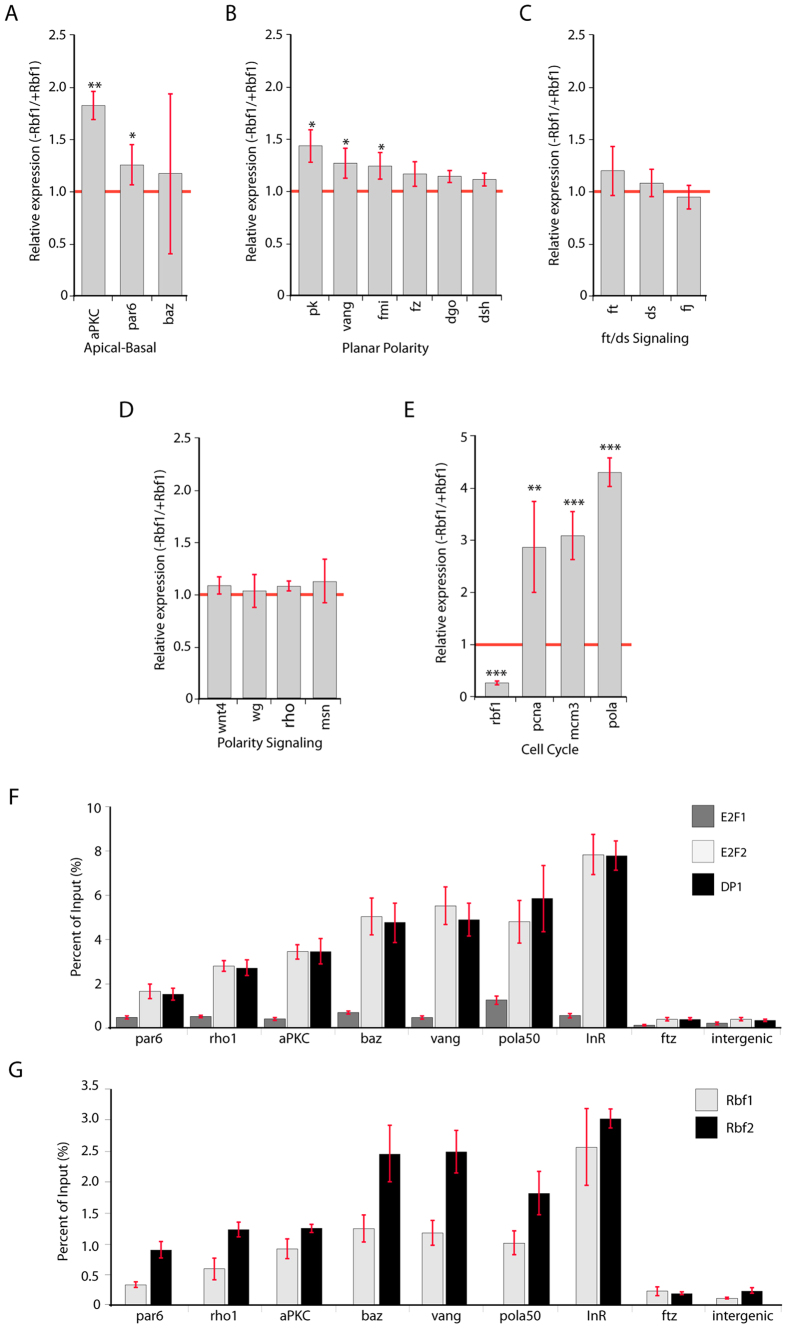
Alterations in Rbf1 levels affect the expression of apical/basal and planar polarity genes. (**A**) Transcript levels of apical/basal polarity genes, from wing imaginal discs that were depleted for Rbf1 by RNAi. *aPKC* and *par6* showed significant upregulation in the RNAi background. (**B**) Planar polarity genes; *pk*, *vang* and *fmi* were modestly but significantly upregulated, while other genes were not significantly affected. (**C**) *ft/ds* pathway genes were not significantly affected (**D**) Expression of polarity-interacting genes was similarly unaffected by Rbf1 depletion. (**E**) *rbf1* mRNA levels were significantly depleted by RNAi treatment, and Rbf1-targeted cell cycle genes *pcna*, *mcm5*, and *DNA polα-50* were upregulated. Red line indicates control expression (Gal4-RNAi). Values for RNAi represent average of five biological replicates (*P ≤ 0.05; **P ≤ 0.01; ***P ≤ 0.001) and error bars represent standard deviations. (**F**) Interaction of E2F1, E2F2, and DP1 transcription factors with promoters of polarity-determining genes assayed by chromatin immunoprecipitation. E2F2 and DP1 were strongly enriched on *aPKC*, *vang*, *baz*, and *rho1*, as well as on positive controls (*inr* and *pol alpha 50*), but not on negative controls (*ftz* and intergenic locus on chromosome 3). (**G**) Rbf2 is also associated with Rbf1 bound promoters on polarity regulating genes. Error bars represent standard deviation. Chromatin was prepared from 12–18 hour embryos as described^8^.

Planar polarity observed in the adult wing is established in the third instar disc^33^. To test whether the observed phenotypes reflect direct transcriptional regulation of key polarity genes by Rbf1, we depleted Rbf1 in third instar larval wing imaginal discs using RNAi and measured the expression levels of target genes. Members of core polarity genes demonstrated significant upregulation. The apical polarity determinant *aPKC* was upregulated 1.8 fold, and *par6*, which encodes a protein that forms a complex with aPKC, showed a smaller but reproducible increase (Fig. 3A). We were unable to determine if *baz*, encoding another *aPKC* complex member, showed a significant change in transcript levels. Half of the genes of *fz* planar polarity pathway (*vang*, *pk* and *fmi*) showed more modest but still highly reproducible upregulation (Fig. 3B). We did not observe significant changes in the expression of *fz*, *dsh*, *dgo*, or members of the *ft*/*ds* pathway (Fig. 3B,C). Transcript levels of additional genes associated with cell polarity signaling (*wnt4, wg, rho1, msn*) were not changed by this treatment (Fig. 3D). Thus, all genes with significant changes in expression showed upregulation, consistent with the role of Rbf1 as a transcriptional repressor. Three of the five upregulated genes (*aPKC*, *vang*, and *par-6*) were previously found to be bound by Rbf1 in our ChIP-seq data, however regulation was not entirely predicted by binding, as three core polarity genes (*fz, dsh, ft*) were not upregulated in this context. As controls, we measured transcript levels of the *rbf1* gene itself and cell cycle genes regulated by Rbf1; RNAi significantly depleted levels of *rbf1*, and we observed corresponding upregulation of *pcna*, *mcm3*, and *DNApol-α50* (Fig. 3E).

RB family proteins interact with target promoters through a variety of mechanisms, chiefly but not exclusively via interaction with E2F transcription factor family members^34^. Some targeted promoters appear to involve additional proteins, such as those of the dREAM complex, or in certain cases non E2F proteins^35^,^36^. Previous ChIP-chip data on whole larvae indicated that *aPKC*, *vang*, *par 6*, *baz*, and *rho1* are bound by Rbf1 and/or E2F2^15^. We had previously noted that Rb targeting tends to be conserved across developmental stages. To provide further information on possible cooccupancy of Rbf and E2F factors on PCP genes, we performed ChIP analysis for these promoters on Rbf1, Rbf2, E2F1, E2F2, and the E2F cofactor DP1. We observed significant enrichment of E2F2 and DP1, but not E2F1, on all of these genes, but not on nonspecific loci (Fig. 3F). We also observed that the Rbf2 protein was also consistently associated with these promoters (Fig. 3G); this protein has a secondary role in regulation of many promoters, hence Rbf1 depletion may be sufficient to affect transcription of the target genes we observed. Here, we used embryonic chromatin; however, binding properties of these proteins appears to be similar in embryos and larvae^15^. We conclude that Rbf1 interaction with the regulated polarity gene promoters uses a canonical E2F-DP1 targeting mechanism, and the regulation of the promoters may in fact resemble that of other signaling pathway genes such as *InR*^36^. Another similarity of protein complexes observed on these polarity gene promoters is the presence of the dREAM complex, a set of DNA-binding factors often associated with RB-bound loci; ChIP-chip data indicates that *aPKC*, *vang*, and *par- 6* are associated with dREAM components in Kc cells^35^.

### Genetic interaction between *Rbf1* and *aPKC*

**Figure 4:**
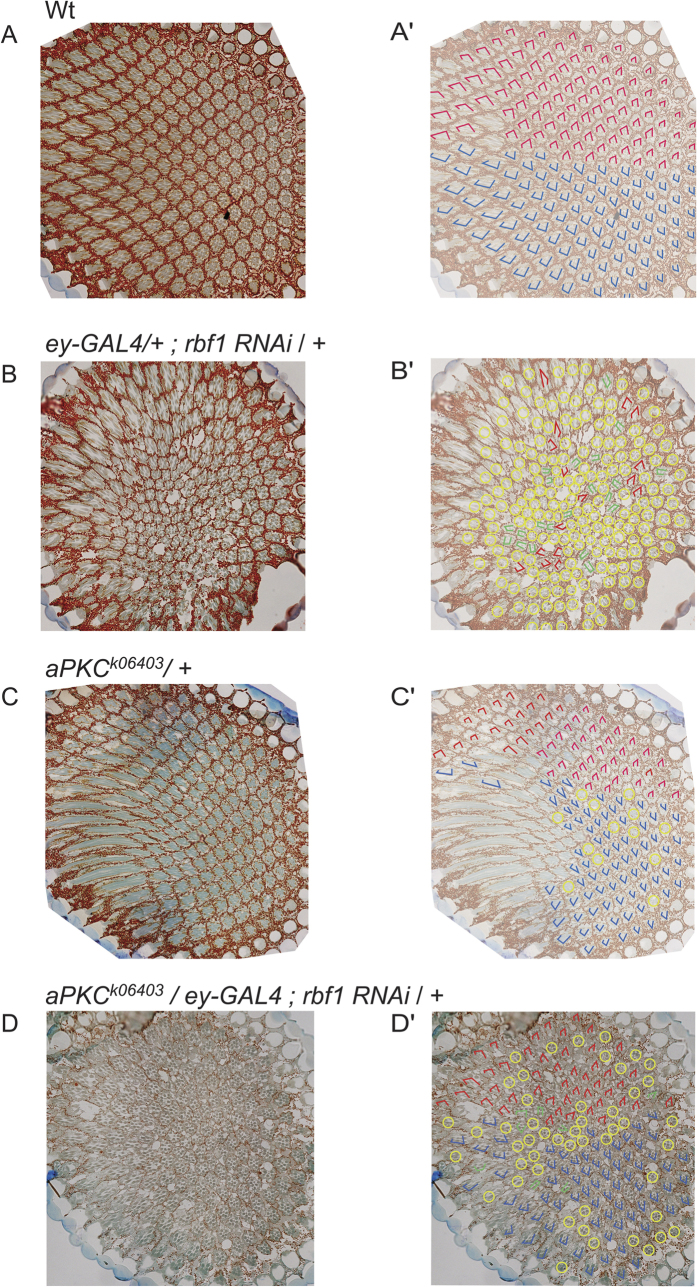
Genetic interaction between *rbf1* and *aPKC* in eye development. (**A,A′**) Section showing imaginal discs of wild-type eye, with markings to indicate dorsal and ventral polarity of photoreceptor cells (red and blue, respectively). (**B,B**′) Rbf1 RNAi eye section showing ommatidia displaying random orientation (red), chirality defects (green), and ommatidia lacking photoreceptor cells (yellow). No equatorial midline could be defined in these sections. (**C,C′**) Eye section of *aPKC* heterozygote showing normal number and orientation of photoreceptors within many ommatidia. A few ommatidia with missing photoreceptors are shown in yellow. (**D,D′**) Eye section of Rbf1 RNAi/*aPKC* heterozygote showing partial rescue. In comparison to *rbf1* heterozygous phenotype (panel B), a large fraction of ommatidia show normal number and correct orientation of photoreceptors with respect to the dorsal/ventral axis. Orientation shows anterior to the right. At least 400 ommatidia were analyzed for polarity defects in multiple sections from four individuals (six for the rescue).

Our data suggests that misexpression of core polarity-regulating genes underlies the polarity defects found in the Rbf1 depleted flies. The largest effect was on the expression of *aPKC*; in the eye, this apical determinant regulates the PCP pathway. Previous work has indicated that upregulation of aPKC produces polarity defects: a constitutively active form of aPKC resulted in misrotated ommatidia and in loss of photoreceptors^19^. To test whether the upregulation of *aPKC* alone was important for the observed phenotypes, we reduced the dose Rbf1 using RNAi in an *aPKC* heterozygous background to test for genetic suppression. In this background, we found an increased number of ommatidia containing the complete complement of photoreceptors (68%). Most ommatidia displayed proper orientation with respect to their A/P and D/V planes with less than 5% percent showing orientation defects and only 28% showing loss of photoreceptors (Fig. 4). The ommatidia of flies that were heterozygous for *aPKC* alone had the normal number and orientation of photoreceptors, with very few ommatidia showing missing photoreceptors. As before, *Rbf1 RNAi* animals showed extensive loss of photoreceptor cells (82%) and rotational defects (18%). The genetic suppression suggests that much, if not all, of the effects observed here are influenced by misregulation of *aPKC*. The possible regulation of other genes by Rbf1 may be more important in other developmental settings, or play a modulatory role. We tried to rescue wing polarity defects in a similar manner by generating a combined *aPKC* heterozygote and Rbf1 RNAi using the *pen-Gal4* driver, however this combination was synthetic lethal. We speculate that the lethality may be a consequence of disruption of neuroblast development, as both aPKC and Rbf1 are functional in this cell type, and the *pen* promoter is active in neuroblasts as well as wing imaginal disc cells^37^,^38^,^39^. Therefore we carried out *Rbf1 RNAi* using *engrailed-Gal4* or *Beadex-Gal4* drivers and observed strong wing polarity defects particularly with the *Beadex* driver. The *aPKC* heterozygous background did not rescue these polarity defects, however (data not shown). Misregulation of other polarity genes may play a more important role in this context. Indeed, tissue specific roles of polarity related genes have been previously observed: *inturned* and *fuzzy* are important for wing polarity but not for eye polarity while *nemo* is a key player in eye but not wing development^40^,^41^.

## Discussion

An important question raised by our study is whether Rbf1 directly coordinates coupling between cell cycle and cell polarity pathways. These two processes are coordinated at some level in diverse systems. For example, in Drosophila neuroblasts, asymmetric division of mother and daughter cells is regulated by the mitotic kinases Polo and Aurora, which are required for both smooth progression through the cell cycle as well as correct localization of aPKC and Numb polarity determinants^42^. Similarly, although RB proteins are not conserved in basal eukaryotes, polarity and cell cycle are nonetheless linked in fission yeast^43^. Coordination can involve simultaneous targeting by transcription regulators, as in Arabidopsis, where the JAG transcription factor coordinately regulates cell cycle and polarity genes in the developing meristem^44^. Our results indicate that Rbf1 is directly influencing the function of polarity factors by regulating their expression levels, providing a mechanism for coordinating the regulation of this pathway with the cell cycle. In this model, Rbf1 would respond to cellular signaling to release from E2F complexes upon the start of S phase in response to cyclin/Cdk phosphorylation. Simultaneous upregulation of cell polarity genes by inactivation of Rbf1 might be important for cells to adjust to an altered surface to volume ratio following cytokinesis. Alternatively, this change in polarity determinants may even be necessary for successful progress through the cell cycle, as observed in neuroblasts, where *apkc* plays a role in both proliferation and polarity^38^. A similar regulatory role for polarity-associated genes *lgl*, *dlg*, and *scrib* on proliferative control has been reported^45^.

Previous studies have indirectly linked mammalian RB to cell polarity; ear-specific deletion of *RB 1* resulted in a cell polarity defect characterized by aberrant inner ear hair cell proliferation and disorganized cilia in the mouse^46^. A role of mammalian RB family proteins in regulating transcription of cell polarity gene promoters has not been described, however, so the mechanism underlying this phenotype has been unknown. We note that among the reported targets of RB and p130 in human lung fibroblasts are the proximal promoter regions of *aPKCΙ*, *fzd2*, *prickle1*, and *celsr1*^6^. Furthermore, among the genes found to be upregulated in these cells upon depletion of RB were *fzd2* and *prickle1*, although the authors did not comment on these findings, and the relevance of this regulation to apical/basal or planar polarity is yet unanswered^6^. Resolving the intertwined roles of RB family proteins on polarity and cell cycle genes will yield a fresh perspective on the mechanisms employed by the RB family proteins.

Our results indicate that transcriptional regulation of polarity-determining genes by RB proteins is an important and evolutionarily conserved process, likely of importance for development in many contexts. Interestingly, a recent report from Knudsen shows that loss of RB in ErbB2- expressing breast cancer cells lead to changes in expression of genes important for maintaining epithelial architecture; mislocaliztion of E-cad and Laminin proteins were also observed in mammary cancer cell cultures, indicative of polarization defects^47^. Regulation of polarity genes by E2F family proteins appears to be another facet of this conserved regulatory mechanism: our analysis of published studies involving perturbations of E2F levels in Drosophila, *C. elegans* and human cells indicates that expression of PCP genes is affected^48^,^49^,^50^. Consistent with this model, null mutants for dDP, the partner of E2F proteins, exhibit dorsal-ventral polarity defects in egg chambers^51^. This subject will be a fruitful area for further inquiry, as disruption of RB-regulated polarity in cancer cells would potentially play an important role in development of metastasis.

## Methods

### Fly stocks and genetics

The *aPKC*^*k06403*^, *UASRbf1* RNAi (Transgenic RNAi Project [TRiP]: HMS03004, Control dsRNA (TRiP: valium 20-Gal4.1) fly lines and *pen *> Gal4 (NP6333), *Bx > Gal4* (stock number 8860), and *en > Gal4* (stock number 30564) stocks were obtained from the Bloomington Stock Center (Bloomington, Indiana). A description of the alleles can be found in Flybase. A second *UASRbf1 RNAi* line was obtained from the Vienna Drosophila Resource Center (VDRC ID 10696). The *ey-Gal4*, *GMR-Gal4*, *ey-Gal4; GMR-Gal4* and *UAS-p35* stocks were kindly provided by Helen McNeill. Males from *UASRbf1* RNAi were crossed to *pen > *Gal4 or *ey* > Gal4 and the progeny were analyzed for PCP defects. More than 100 wings were analyzed for wing defects and for ommatidial rotation defects n > 400 ommatidia were analyzed. Cuticle refraction microscopy was performed to obtain images of wing membrane topography^32^. Adult eye sections were prepared as described in Wolff, 2000^52^. A minimum of 4 individuals were sectioned from each genotype and analyzed for ommatidial defects. For the genetic suppression studies, we generated flies of the genotype *aPKC*^*k06403*^/Gla-Bc; U*ASRbf1 RNAi* or Control Gal4.1/TM6 Tb^1^ and crossed them to homozygous *pen * > Gal4 or *en >  Gal4*/*SM2Cyo* or *Bx* > *Gal4* or *ey* > Gal4/SM2 Cyo flies. *aPKC*^*k06403*^/*pen * > Gal4; U*ASRbf1 RNAi*/TM6 Tb^1^ was lethal, precluding analysis of adult wing, however we were able to recover *aPKC*^*k06403*^/*ey * > Gal4; U*ASRbf1 RNAi*/TM6 Tb^1^ animals, which were analyzed for ommatidial rescue. Five to ten individuals from at least two completely separate genetic crosses and more than 400 ommatidia were analyzed for all experiments. We analyzed two individuals from *GMR-Gal4* > U*ASRbf1 RNAi* (RNAi line obtained from Bloomington) and counted greater than 300 ommatidia for polarity defects.

### Quantitative PCR

Wing imaginal discs were dissected from third-instar larvae, and total RNA was isolated according to the protocol^53^ using TRIzol (Invitrogen) followed by RNeasy Mini kit (Qiagen) for cleanup. 300 ng of total RNA was converted to cDNA using High Capacity cDNA Reverse Transcription kit (Applied Biosystems). The resulting cDNA was diluted 1:15 and 3 μl was used for PCR in a 20 μl reaction mixture using SYBR green PCR Master Mix (Applied Biosystems). qPCR was performed on five biological replicates and the fold change in gene expression was calculated based on the 2^−ΔΔC^_T_ method and normalized to *rp49* gene expression. Sequences for primers used for gene expression analysis are available upon request.

### Chromatin Immunoprecipitation

Chromatin was prepared from 12–18 hour embryos following the protocol described in Acharya *et al.* 2012^8^. Immunoprecipitations were carried out using Rbf1 and Rbf2 antibodies^43^. E2F and DP antibodies were gifts from Dr. Nicholas Dyson (Harvard Medical School). qPCR using SYBR green PCR Master Mix (Applied Biosystems) was performed on two biological replicates, and results in Fig. 3F,G show averages of these experiments. Sequences for primers used for PCR were situated under predicted Rbf1 binding areas in promoter proximal regions, except for negative control genes, and are available upon request. The intergenic negative control represents region on Drosophila chromosome 3L (*chr 3L: 13736222–13736322*).

### Statistical Analysis

Data represents mean ± SD. P values were determined by unpaired Student’s t test.

## Additional Information

**How to cite this article**: Payankaulam, S. *et al.* Regulation of cell polarity determinants by the Retinoblastoma tumor suppressor protein. *Sci. Rep.* **6**, 22879; doi: 10.1038/srep22879 (2016).
